# Decline in health-related quality of life and foot and ankle patient reported outcomes measures in patients with haemophilia and ankle haemarthropathy

**DOI:** 10.1186/s13047-023-00611-5

**Published:** 2023-03-10

**Authors:** Richard A. Wilkins, Heidi J. Siddle, Graham J. Chapman, Elizabeth Horn, Rebecca Walwyn, Anthony C. Redmond

**Affiliations:** 1 Leeds Institute of Rheumatic and Musculoskeletal Medicine, University of Leeds, Leeds, UK; 2 Leeds Haemophilia Comprehensive Care Centre, Leeds Teaching Hospitals NHS Trust, Leeds, UK; 3 School of Sport and Health Sciences, University of Central Lancashire, Preston, UK; 4 Clinical Trials Research Unit, Leeds Institute of Clinical Trials Research, University of Leeds, Leeds, UK; 5 NIHR Leeds Biomedical Research Centre, Leeds Teaching Hospitals NHS Trust, Leeds, UK

**Keywords:** Impact, Ankle, Haemarthrosis, Haemarthropathy, Health-related quality of life

## Abstract

**Background:**

Haemophilia is an X-linked recessive genetic disorder characterised by bleeding within soft tissue and joints. The ankle is disproportionally affected by haemarthropathy when compared to the elbows and knees; reported as the most affected joints in patients with haemophilia. Despite advances in treatment, patients still report ongoing pain and disability, however, the impact has not been evaluated, nor has the effect on health-related quality of life (HRQoL) or foot and ankle patient-reported outcome measures (PROMs). The primary aim of this study was to establish the impact of ankle haemarthropathy in patients with severe and moderate haemophilia A and B. Secondly to identify the clinical outcomes associated with a decline in HRQoL and foot and ankle PROMs.

**Methods:**

A cross-sectional multi-centre questionnaire study was conducted across 18 haemophilia centres in England, Scotland and Wales with a recruitment target of 245 participants. The HAEMO-QoL-A and Manchester-Oxford Foot Questionnaire (MOXFQ) (foot and ankle) with total and domain scores measured impact on HRQOL and foot and ankle outcomes. Demographics, clinical characteristics, ankle haemophilia joint health scores, multi-joint haemarthropathy and Numerical Pain Rating Scales (NPRS) of “ankle pain over the past six months” were collected as a measure of chronic ankle pain.

**Results:**

A total of 243 of 250 participants provided complete data**.** HAEMO-QoL-A and MOXFQ (foot and ankle) total and index scores indicated worse HRQoL with total scores ranging from a mean of 35.3 to 35.8 (100 best-health) and 50.5 to 45.8 (0 best-health) respectively. NPRS (mean (SD)) ranged from 5.0 (2.6) to 5.5 (2.5), with median (IQR) ankle haemophilia joint health score of 4.5 (1 to 12.5) to 6.0 (3.0 to 10.0) indicating moderate to severe levels of ankle haemarthropathy. Ankle NPRS over six months and inhibitor status were associated with decline in outcome.

**Conclusions:**

HRQoL and foot and ankle PROMs were poor in participants with moderate to severe levels of ankle haemarthropathy. Pain was a major driver for decline in HRQoL and foot and ankle PROMs and use of NPRS has the potential to predict worsening HRQoL and PROMs at the ankle and other affected joints.

## Background

Haemophilia is an X-linked recessive genetic disorder characterised by bleeding in soft-tissue and joints caused by an absence or reduction in circulation clotting factor needed to maintain haemostasis [1]. The two most common types of this rare disease are haemophilia A (1:5000 people) and B (1:30,000 people), the absence of factor VIII and VIX respectively [2, 3]. Haemophilia is further characterised but severity as mild (> 0.05—< 0.24 IU/mL or more than 5%) moderate (0.01–0.05 IU/mL or 1 to 5%) and severe (< 0.01 IU/mL or less than 1%) [2].

Multi-joint haemarthropathy is an inherent clinical feature of severe haemophilia and moderate haemophilia with a more severe phenotype [1, 4]. Severe haemophilia A is reportedly associated with worsening levels of joint haemarthropathy when compared to patients with haemophilia B and moderate haemophilia who report lower incidence and severity of haemarthrosis and haemarthropathy [4, 5]. Changes in treatment recommendations aimed at reducing the clinical burden of haemarthrosis in moderate haemophilia have been recently published by the United Kingdom Haemophilia Doctors Organisation and promote the initiation of haemostatic management with prophylaxis treatment regimens if the patient experiences haemarthrosis or clinically significant bleeding [6, 7].

Despite improvement in primary and secondary prophylaxis treatment with novel factor and non-factor treatment regimes, musculoskeletal complications of repeated and historical haemarthrosis remain a long-term health issue resulting in significant levels of impairment and pain [8, 9]. The ankle is particularly problematic regarding the development of joint damage with disproportionate levels of haemarthropathy when compared to the other most affected joints, the knees and elbows [10]. A single significant or repeated minor incidence of haemarthrosis leads to synovial hypertrophy, haemosiderin deposition and eventual haemarthropathy [11–13]. Highly vascularised synovium and changes to the composition of articular cartilage compositions reduce the ability to dissipate compressive and shear forces. The combination of synovitis and functional joint changes leads to articular cartilage degeneration, bone damage, loss of joint space and chronic end-stage haemarthropathy [14–16]. Changes to ankle joint structure and function have been attributed to loss of muscular control associated with early episodes of haemarthrosis exposing the ankle joint to high forces and shearing during activities of daily living, however no definitive evidence has been cited [17–19].

The physical and mental burden of haemophilia is great when compared to the general population [20]; changes to joint status is a particular issue in patients with haemophilia who report periods of pain and disability. Health-related quality of life (HRQoL) is linked to severity of disease; patients with severe haemophilia are the most affected compared with those with mild haemophilia who report having the best HRQoL [21, 22].

The ankle joint is often cited as the main site of pain and haemarthropathy however, little has been reported on how ankle haemarthropathy contributes to overall musculoskeletal health, HRQoL and foot and ankle specific patient-reported outcome measures (PROMs) [23–26]. Similarly, it is not understood how disease characteristics and clinical measures of ankle joint health, pain and arthropathy contribute to outcomes. It was therefore the aim of this study to report the current impact of ankle haemarthropathy on HRQoL, foot and ankle PROM and its relationship to other clinical measures of joint pain and joint health.

## Methods

### Study aims

To report the impact of ankle haemarthropathy on HRQoL and foot and ankle PROMs in UK patients with moderate and severe haemophilia. The study also aims to identify clinical associations of decline in this cohort.

Ethical approval was obtained on 24th January 2017 (IRAS: 206,141, REC ID: 16/LO/2251, R&D: PD16/227). Recruitment commenced on 13^th^ April 2017 and ended on 31st August 2019. Recruitment was undertaken at 18 sites across England, Scotland and Wales, with support from the NIHR clinical research network (non-malignant haematology).

### Study material

A cross-sectional multi-centre questionnaire consisting of two sections was distributed to participating haemophilia comprehensive care centres and haemophilia treatment centres (Appendix A). Section A contained the validated HRQoL measure the HAEMO-QoL-A and the foot and ankle specific outcome measure the MOXFQ (foot and ankle). Participants provided demographic information, detail of disease characteristics (haemophilia type, severity, and inhibitor status), and treatment (product, dose, pain medication). Ankle pain status over the past six months was capture using a numerical pain rating scale (NPRS). Participants were asked “how painful has your ankle been over the past six months?”, 0 = No pain to 10 = Pain as bad as you can imagine [27]. Section B was completed by the centre nurse, Allied Health Professional (AHP) or consultant haematologist and confirmed details of disease characteristics, treatment product and a recent haemophilia joint health score for the ankles only.

### Study population

A recruitment target of 245 was set to allow the mean HAEM-QoL-A to be estimated to be within ± 2.5 units of the measurement scale, assuming a between the patient standard deviation of 16.96 [28]. Inclusion criteria consisted of patients aged 18 and over with moderate and severe haemophilia A or B with a consultant diagnosis of ankle haemarthropathy confirmed by x-ray or magnetic resonance imaging. Participants were identified at their associated haemophilia comprehensive care centre or haemophilia treatment centre by a Nurse, AHP or consultant haematologist.

### Statistical analysis

To report associations between participant disease status and HEAMO-QoL-A and MOXFQ (foot and ankle) total score and domain data, the outcomes were entered into a linear regression model where the effect of haemophilia type, severity and treatment regime were entered separately as predictors of decline. Sensitivity analysis was undertaken using stepwise regression models to determine whether clinical measures of pain, inhibitor status, and ankle haemophilia joint health score were associated with decline in HRQoL and foot and ankle PROMs subscale and total scores. Data are reported as mean and standard deviation, non-parametric data are presented and median and interquartile range (25^th^ and 75^th^ percentiles).

## Results

At the close of recruitment, 250 response sets had been received. Data from seven patients were excluded from the analysis due to the incompleteness of the main outcome measures (HAEMO-QoL-A and MOXFQ), leaving 243 for analysis.

### Patient characteristics

Patient demographics and disease characteristics are presented in Table 1. Overall, 214 (88.1%) patients had severe and 29 (11.9%) had moderate haemophilia. Severe haemophilia A participants made up the largest proportion (*n *= 184) of the cohort. Age, height and weight were similar across haemophilia type and severity. Body mass index (BMI) was similar across haemophilia types, with the majority of patients classed as over-weight. Patients receiving standard half-life (SHL) clotting factor concentrate treatment were exclusive to haemophilia A, with 107 (45.3%) patients using SHL products. Extended half-life products were used by 88 (37.38%) and 32 (13.55%) patients with haemophilia A and B respectively. Nine (3.8%) patients used a bispecific monoclonal antibody**,** which at the time of this study, were used exclusively in the management of haemophilia A with a current inhibitor.

**Table 1:** Patient demographics and disease characteristics

Severity and treatment Mean (SD)		Haemophilia A	Haemophilia B
**Severe**	**Number**	184/ 75.7%	30/ 12.3%
	**Age**	42.4 (13.1)	47.3 (11.5)
	**Prophylaxis/ on-demand treatment**	164 (67.5%)/ 20 (8.2%)	27 (11.1%) / 3 (1.2%)
	**Current inhibitor**	16/ 6.6%	1/ 0.4%
	**Height (cm)**	176.9 (7.4)	177.6 (7.3)
	**Weight (Kg)**	84.1 (18.3)	89.4 (19.9)
	**BMI (Kg/m**^**2**^**)**	26.7 (5.3)	27.8 (7.1)
**Moderate**	**Number**	25/ 10.3%	4/ 1.7%
	**Age**	48.9 (15.9)	46.6 (18.1)
	**Prophylaxis/ on-demand**	11 (4.5%)/ 14 (5.8%)	3 (1.2%)/ 1 (0.4%)
	**Current inhibitor**	1/ 0.4%	0
	**Height (cm)**	177.2 (7.9)	180.6 (9.6)
	**Weight (Kg)**	85.0 (16.2)	98.4 (18.9)
	**BMI (Kg/m**^**2**^**)**	26.8 (4.4)	30.1 (4.5)

*BMI* Body Mass Index, *SD* Standard deviation, *IQR* Interquartile range (25 and 75 percentile), † Only three participants reported treatment dose therefore no IQR reported, ‡ Data not reported

### Patient HRQoL and ankle specific PROM

Patient questionnaire data and clinical measures are presented in Table 2. Individual domain and total scores of the HEAMO-QoL-A were generally poor (100 = best health) across severities indicating low HRQoL. The foot and ankle specific PROMs the MOXFQ index scores were high (0 = best health) with individual domain scores (Table 2) that indicate worsening pain and function.

**Table 2:** Patient Questionnaire and clinical measures

*Outcomes*	*Severe haemophilia (n* = *214)*	*Moderate haemophilia (n* = *29)*
***HAEMO-QoL-A* Total index Score**^**†**^	***35.3 (14.9)***	***35.8 (17.4)***
* Physical function*	*48.6 (11.6)*	42.3 (13.9)
* Role Function*	*31.3 (23.0)*	26.1 (23.3)
* Worry*	*27.0 (25.5)*	25.8 (26.0)
* Consequence of bleeding*	*29.4 (23.1)*	31.7 (26.5)
* Emotional impact*	*56.5 (18.6)*	59.1 (19.7)
* Treatment concerns*	*23.6 (25.0)*	30.1 (30.5)
***MOXFQ Total score***	***50.5 (24.2)***	***45.8 (24.7)***
* Social*	*6.9 (4.0)*	*5.3 (4.0)*
* Pain*	*9.9 (4.9)*	*9.5 (5.1)*
* Walking/ standing*	*16.4 (8.0)*	*14.5 (8.0)*
***NPRS average ankle pain in the past 6 months***	*5.0 (2.6)*	*5.5 (SD,2.5)*
Ankle HJHS (left/ right)*	*6.0 (3.0; 10.0)/ 5.0(2.0; 9.0)* *HA 6.0 (3.0;10.0)/ 6.0 (2.0;9.0)* HB 5.0 (2.0; 9.0)/ 3.0 (0;6.0)*	*4.5 (1; 12.5/ 3.0 (0 9.5)*
***Ankle haemarthropathy (one/both)***	*103 (48.1%)/ 104 (48.6%)*	*16 (55.2%)/ 13 (44.8%)*
***Elbow haemarthropathy (one/both)***	*77 (36%)/ 26 (12.1%)*	*7 (24.1%)/ 3 (10.3%)*
***Knee Haemarthropathy (one/ both)***	*55 (25.7%)/ 15 (7%)*	*8 (27.6%)/ 4 (13.8%)*

*NPRS* Nunmerical pain rating scale, *SD* Standard deviation, *IQR* Interquartile range (25 and 75 percentile)

### Ankle pain and management

Ankle pain severity experienced over six months (Table 2) were consistent across haemophilia, type and severity. Only 233 patients provided details of pain medication use, with 56.2% (*n* = 131) indicating they did not use regular pain medication. In the remaining 43.7% (*n* = 102) patients paracetamol and COX2 inhibitors were the most commonly used pain medications.

### Regression analysis

Regression analysis of haemophilia disease and treatment characteristics were not independently associated with the decline in total or domain scores of the HEAMO-QoL-A and MOXFQ. Stepwise regression analysis of the clinical variables associated with the HEAMO-QoL-A and MOXFQ total scores excluded ankle haemophilia joint health score (left and right), treatment IU/kg and treatment product from the final model. Ankle NRPS and inhibitor status accounted for 52% of the R-Square for HAEMO-QoL-A total scores. NPRS and inhibitor status accounted for 53.7% of the R-Square for Total MOXFQ. Sensitivity analysis of the total and subscales of the HEAMO-QoL-A and MOXFQ are presented in Table 3. NPRS of ankle pain over six months was found to be independently associated with all total and individual domains for both outcome measures. Inhibitor status was similarly associated with decline.

**Table 3:** Stepwise regression analysis final model of the total and individual domains of the HAEMO-QoL-A and Manchester Oxford foot and ankle Questionnaire (MOXFQ)

Outcome measure	Variable	Unstandardized Coefficients B	Sig	95% Confidence Interval	
				**Lower bound**	**Upper bound**
**Haemo-Qol-A total score**	NPRS six months	0.76	< 0.001	0.56	0.98
	Inhibitor status	4.14	0.001	1.68	6.55
Physical function	NPRS six months	0.05	0.003	0.02	0.09
	Factor product	-0.50	0.009	-0.08	-0.01
	On demand treatment	0.31	0.013	0.07	0.56
Role function	NPRS six months	0.22	< 0.001	0.16	0.28
	Inhibitor status	0.85	0.018	0.15	1.55
Worry	NPRS six months	0.24	< 0.001	0.18	0.31
	Inhibitor status	0.79	0.041	0.03	1.55
Bleeding	NPRS six months	0.19	< 0.001	0.12	0.25
	Inhibitor status	1.04	0.005	0.30	1.79
Emotion	NPRS six months	-0.11	< 0.001	-0.162	-0.06
**MOXFQ (foot and ankle) total score**	NPRS six months	6.84	< 0.001	5.88	7.80
	Inhibitor status	11.39	0.048	0.12	22.65
Walking/ standing	NPRS six months	1.85	< 0.001	1.50	2.20
	HJHS right	0.23	0.021	0.04	0.43
Pain	NPRS six months	1.40	< 0.001	1.22	1.59
Social	NPRS six months	1.05	< 0.001	0.89	1.24
	Inhibitor status	2.85	0.011	0.67	5.04

## Discussion

The primary aim of this study was to identify the impact of ankle haemarthrosis and haemarthropathy in patients with moderate and severe haemophilia. This large multicentre study has identified that across the UK, patients with moderate and severe haemophilia experience poor HRQoL and foot and ankle PROM.

The impact of multi-joint haemarthropathy has previously been associated with severity. Patients with severe haemophilia report worse outcomes than moderate and mild disease [21, 22]. Higher levels of disability are associated with the change in joint structure and function at the ankles, knees and elbows leading to higher levels of disability, increased risk of bleeding, pain and reduced HRQoL [25, 29, 30]. Patients with moderate and mild disease have been reported to be less affected by the incidence of bleeding, complication and joint damage [21, 22]. Similarly, in patients with haemophilia B frequency, intensity and levels of haemarthropathy are lower than in patients with haemophilia A [4]. This was not the case in this study, with moderate haemophilia and haemophilia B equally affected indicating that in the presence of ankle haemarthropathy HRQoL is poor. Whilst the sample of haemophilia B and moderate haemophilia were low (*n* = 34 and *n* = 29 respectively) the findings are similar to that of De Juili et al. (2014) who in a much larger sample of patients (*n* = 75) found that despite few complications of bleeding, a proportion of moderate haemophilia patients was more severely affected by haemarthropathy [31]. Haemophilia B is also reported to have a lower incidence of bleeding and less joint damage [4]. Whilst these study results are interpreted with caution; it is apparent that when the physical manifestation at the ankle report moderate to severe haemarthropathy the impact on HRQoL is equivalent across disease types and severity. It remains unclear as to what point arthropathic joint changes lead to decline in HRQoL.

The clinically detectable levels of haemarthropathy, measured using the haemophilia joint health score indicate advancing haemarthropathy across both disease severities. There is no consensus as to the level of haemarthropathy indicated by the haemophilia joint health score, however radiological compassions using the Pettersson score a radiological measure of joint damage in haemophilia suggests haemophilia joint health score of 5.0 to 6.0 correlates with moderate to severe levels of haemarthropathy [32]. The level of haemarthropathy in this study varied across disease characteristics (Table 2), however foot and ankle PROM scores were again similar between haemophilia types, severity and treatment regimes. Total and individual domains of walking/ standing, pain and social interaction were all poor and similar to patients with ankle osteoarthritis (OA) awaiting ankle fusion surgery and total ankle replacement indication chronic ankle pain and disability. There are a limited number of studies that have directly reported foot and ankle PROM in haemophilia [33]. To date, only two studies of footwear and foot orthoses interventions have used foot and ankle outcome measures, the foot function index (FFI) and the FFI revised, indicate moderate levels of haemarthropathy correlated with moderate impact [23, 24]. In this study participants had higher ankle joint haemophilia joint health score that correlates with higher levels of joint disease. This may therefore explain why our HRQoL and foot and ankle PROMs are more severely affected than previously sighted haemophilia cohorts with ankle haemarthropathy.

Ankle pain was the most impactful feature across all haemophilia disease characteristics. NPRS ankle pain ranged from 4.9 to 5.5 across the cohort with similar levels reported in studies of severe haemophilia. A large US survey of pain reported NPRS average mean (SD) persistent pain of 4.32/10 (SD, 2.53) in moderate and 4.25/10 (SD, 1.90) in severe haemophilia; patient-reported pain was also the most significant association with decline in HRQoL [34]. Although the level of haemarthropathy was not reported and the healthcare systems of countries differ, scores were slightly lower than this studies cohort, suggesting that the data is representative of severe haemophilia in both acute and chronic pain driven by synovitis, and chronic joint disease [34].

The NRPS “average pain in your ankle over the past six months” has been based on the Initiative on Methods, Measurement, and Pain Assessment in Clinical Trials chronic pain guidance and appears to be associated with decline for both HRQoL and foot and ankle PROMs [35]. This clinical measure of pain, easily administered in clinical practice has, therefore, the potential to be used to identify decline in outcomes and identify when further interventions may be required, such as the modification of pain management, physical therapy and psychology.

Pain management, despite high levels of patient-reported chronic pain, was poor with less than half the cohort using regular pain medication, with 56% of patients not use pain medication [30]. The complexities of pain in haemophilia are acknowledged where patients are known to experience pain differently, develop coping strategies such as exercise, massage, physical therapy and distraction techniques by ignoring pain to combat symptoms [9, 36]. Pain management in the haemophilia community is regarded as poor with 40% of patients reporting difficulties in obtaining appropriate pain management from their healthcare provider. Survey of pain management of haemophilia adults aged 40 and 65 years with multi-joint haemarthropathy have reported lack of access to pain relief for the majority of childhood where joints are more prone to acute painful episodes of haemarthrosis [37]. Coping with high levels of pain and management without pain relief is therefore synonymous with chronic haemarthropathy [37]. Study findings raise valid questions as to the pain in haemophilia and the need for target pharmacological and non-pharmacological interventions especially in the presence of multi-joint haemarthropathy where the ankle joint has been reported to account for 45% of all joint pain [25]. A recent systematic review of pain management showed physiotherapy interventions lacking the methodological trial designs to make any conclusive recommendations for pain management [38]. Similarly in the management of ankle haemarthropathy, there is low-quality evidence that the use of foot orthoses and footwear interventions reduce pain, however, there is no conclusive evidence to change clinical management and guidance [39].

The development of inhibitors is a major complication of haemophilia management both physically and physiologically [36, 40, 41]. The presence of an inhibitor is associated with increased levels of joint arthropathy, chronic pain and long periods of hospitalisation, absenteeism for work and decline in QoL when compared to non-inhibitor patients [36]. It is therefore without surprise that this study has identified inhibitor status as being associated with decline in HRQoL and foot and ankle outcomes. The results of this study highlight the need to assess the non-physical contributions of inhibitor status and the need to closely monitor inhibitors to prevent or delay the development of ankle haemarthropathy.

### Limitations

The findings of this study and the associations with decline in HRQoL and foot and ankle PROMs need to be replicated in another sample such as patients changing to novel factor and non-factor treatment therapies to confirm whether ankle haemarthropathy is a major contributor to decline in the haemophilia population. The small sample size of moderate and haemophilia B patients is acknowledged as a limitation of this study and their results should be interpreted with caution. The small sample size highlights the difficulties in undertaking research in rare diseases and obtaining an adequate sample size of subgroups within the selected population. Whilst these samples are small, this study provides focus to the emerging issue of the impact of ankle haemarthropathy outside of severe haemophilia A.

## Conclusion

In the presence of ankle haemarthropathy HRQoL is poor and foot and ankle PROMs are significantly affected. Pain appears to be a major driver of the decline in outcomes, with patients with ankle haemarthropathy having high levels of chronic pain. The use of an NPRS for average pain over six months is associated with decline in HRQoL and identify the non-physical burden of the patient’s disease at the ankle and other commonly affected joints. Use of a NPRS of average ankle pain over six month period may be used to assess current pain status in clinical practice and direct the use of HRQoL and foot and ankle PROMs. Further research is needed to provide robust evidence in clinical practice for the use of pharmacological and non-pharmacological interventions that may improve pain and prevent the decline in ankle joint health across haemophilia.

## Data Availability

The datasets used and/or analysed in this study are available from the corresponding author on reasonable request.
